# Alendronate Effect on the Prevention of Bone loss in Early Stages of Ankylosing Spondylitis: A Randomized, Double-Blind, Placebo-Controlled Pilot Study

**DOI:** 10.5812/ircmj.18022

**Published:** 2014-06-05

**Authors:** Alireza Khabbazi, Hamid Noshad, Sevil Gafarzadeh, Mehrzad Hajialiloo, Susan Kolahi

**Affiliations:** 1 Connective Tissue Diseases Research Center, Tabriz University of Medical Sciences, Tabriz, IR Iran; 2 Chronic Kidney Disease Research Center, Tabriz University of Medical Sciences, Tabriz, IR Iran; 3 Department of Internal Medicine, Tabriz University of Medical Sciences, Tabriz, IR Iran

**Keywords:** Spondylitis, Ankylosing, Bone Density, Alendronate

## Abstract

**Background:**

Ankylosing spondylitis (AS) is an inflammatory rheumatic disease that leads to a progressive ankylosis of vertebras and ossification of paravertebral ligaments. Bone loss and osteoporosis are amongst the important complications of AS, treatment of which is a challenging issue.

**Objectives:**

This study aimed to clarify the effect of alendronate on the prevention of bone loss in patients with early AS.

**Patients and Methods:**

In a randomized, double-blind, placebo-controlled study, 24 patients with early stages of AS were recruited in Emam Reza Hospital, Tabriz University of Medical Sciences. The diagnostic criteria of early AS were Schober’s index ≥ 5, normal hip joint in pelvic radiography, and absence or rarity of syndesmophytes in spine radiography (Taylor index ≤ 1). The participants were randomly allocated to the treatment and control groups and received 70 mg/week of alendronate and the same dose of placebo, respectively, for 12 months. Before and 12 months after the intervention, bone densitometry was performed from lumbar and pelvic region using the dual-energy X-ray absorptiometry (DEXA) method with Hologic QDR model instrument. Patients, physicians who prescribed the medications and those who interpreted the outcomes, and densitometry technicians were unaware of the assigned medication to each patient. Both groups received supplemental calcium (1000 mg/day) and vitamin D (400 mg/day).

**Results:**

After 12 months of treatment, hip and lumbar bone mineral density differences were not statistically significant between study groups (P = 0.061 and P = 0.112, respectively). No case of clinically apparent vertebral and nonvertebral fracture were observed in the treatment and control groups.

**Conclusions:**

Our results suggested that applying alendronate was ineffective in preventing bone loss in patients with early stages of AS.

## 1. Background

Ankylosing spondylitis (AS) is an inflammatory rheumatic disease that is characterized by progressive ankylosis of vertebras and ossification of paravertebral ligaments and vertebral discs, which lead to the increased rigidity of the spine. The prevalence rate of AS is 7.4 to 31.9 per 10000 population (1, 2). Bone loss and osteoporosis are amongst the important complications of AS. Fractures, especially fractures of cervical spines, are more frequent in patients with AS than normal people (1.4%-58%) (3). Etiology of spinal fractures in AS are vertebras ankylosis and osteoporosis. Bone inflammation in AS leads to severe changes in bone turnover, which is the main cause of osteoporosis and the susceptibility to fractures (4). In a radiographic study, osteoporosis was detected in more than half of the patients with AS (5). The frequency of osteoporosis in AS was 18.7% to 62% in other similar studies (6-8). Bone loss is common in patients with long duration of AS; however, the prevalence of decreased bone mineral density (BMD) in patients with short disease duration is also high (9). In a systematic review, van der Weijden et al. showed that the prevalence of low BMD in the early stages of AS (disease duration < 10 years) were 51%-54% for the femoral neck and the lumbar spine, respectively (10). Davey-Ranasinghe et al. revealed that the rate of the BMD reduction in AS was 2.2% per year, while in normal men it is about 0.7% per year (11). Ghozlani et al. showed that the frequency of spinal fracture in patients with and without osteoporosis is 29.6% and 11.1%, respectively (12). Although osteoporotic fracture is more common in prolonged AS, it may be seen in early stages too (13); therefore, osteoporotic fracture might be the first presentation of AS (14). Vertebral fractures are associated with pain, deformity, and sometimes neurologic problems (15). Diagnosis of vertebral fracture may be missed due to the pain of the disease (16). Bone densitometry of hip with dual-energy X-ray absorptiometry (DEXA) method is often used to diagnose osteoporosis in patients with AS (4). In AS, hip density is low and will reduce over time. Some studies showed that hip osteopenia is seen in 72% to 93% of the patients with AS (17). In early AS, density of vertebral spines reduces, but it increases over time due to formation of the syndesmophytes, which falsely increases vertebral bone content (6). Therefore, measuring lumbar spine density is accurate only in the early stages of AS.

Unlike patients with rheumatoid arthritis (RA), pharmaceutical treatment of osteoporosis in patients with AS is not yet a common practice. Data in support of the efficacy of osteoporosis treatment in AS are scarce. Bisphosphonates accumulate at the sites of increased bone turnover, inhibit bone resorption by inducing osteoclast apoptosis, and thereby increase bone density (18). Bisphosphonates were used widely for the treatment of osteoporosis in patients with AS, but there is not enough controlled study (16) regarding their effects on all forms of osteoporosis and their antiresorptive and anti-inflammatory effects (19). Current data show a continual demineralization in AS, which starts in the early stages of the disease; therefore, it is logical to prevent osteoporosis in early AS. No study was performed, to the best of our knowledge, to assess the efficacy of bisphosphonates in the prevention of bone loss and osteoporosis in early AS.

## 2. Objectives

Due to the high prevalence of osteoporosis and fractures in AS, we decided to design a pilot clinical trial to assess the effect of alendronate in preventing bone loss in early stages of AS. 

## 3. Patients and Methods

This randomized, double-blind, placebo-controlled study was conducted in Emam Reza Teaching Hospital, Tabriz University of Medical Sciences (TUOMS), Tabriz, Iran, from July 2011 to October 2013. The study was approved by the Ethics Committee of the TUOMS and was registered with the Iranian Registry of Clinical Trials (IRCT) with the registration number of IRCT201212206975N3. All the patients referred to the outpatient clinic of Connective Tissue Diseases Research Center of TUOM who fulfilled the Assessment in Spondyloarthritis International Society (ASAS) classification criteria for axial spondyloarthropathies were eligible for our study (20). We used simple random allocation. All the patients in the early stage of AS with normal BMD or minimal reduction in bone density (T-score ≤ −1.5) were enrolled in this study. The criteria for early stage of AS were Schober’s index ≥ 5, normal hip joint in pelvic radiography, and absence or rarity of syndesmophytes in spine radiography (Taylor index ≤ 1). The exclusion criteria were previous history of spinal fracture, bisphosphonates and corticosteroids administration, pregnancy and lactation, and other conditions that might affect BMD like hypothyroidism, hyperthyroidism, osteomalacia, hyperparathyroidism, diabetes mellitus, and liver or kidney failure. Patients enrolled in this study after signing the written informed consents. Disease activity and axial status were evaluated by the Bath ankylosing spondylitis disease activity index (BASDAI) (21) and Bath ankylosing spondylitis metrology index (BASMI) (22), respectively. These patients were randomly allocated to two groups with Randlist software (DatInf GmbH, Tubingen, Germany). The treatment group received 70 mg/week of alendronate (Ostomod, Modava Company, Iran) for 12 months and the control group received placebo with the same dose for the same period of time. Both groups received 1000 mg of calcium and 400 mg of vitamin D daily. Bone densitometry was performed with Hologic QDR model instrument (Hologic Inc., Waltham, MA, USA) before and 12 months after the intervention. Patients, physicians who prescribed or assessed the final outcome, and densitometry technicians were unaware of the type of the therapy. In order to control inflammation, we used anti-inflammatory dose of nonsteroidal anti-inflammatory drugs (NSAID) with in both groups. Patients visited every two months and radiography was performed in the presence of the clinical signs of osteoporotic fracture. In this study, primary and secondary endpoints were BMD changes and clinical spinal and nonspinal fractures, respectively.

### 3.1. Statistics

A descriptive study was performed using SPSS v. 15.0 (SPPS Inc., Chicago, IL, USA). We used ANOVA, Chi square, and Fisher’s exact test for analyzing data. In this study, P < 0.05 was considered statistically significant.

### 3.2. Ethical Points

Alendronate is a bisphosphonate and its main side effect is gastrointestinal complication. It is observed only in 5% of patients and is reversible after discontinuing. All the patients in this study signed a written consent and were aware of the complications.

## 4. Results

**Figure 1.**
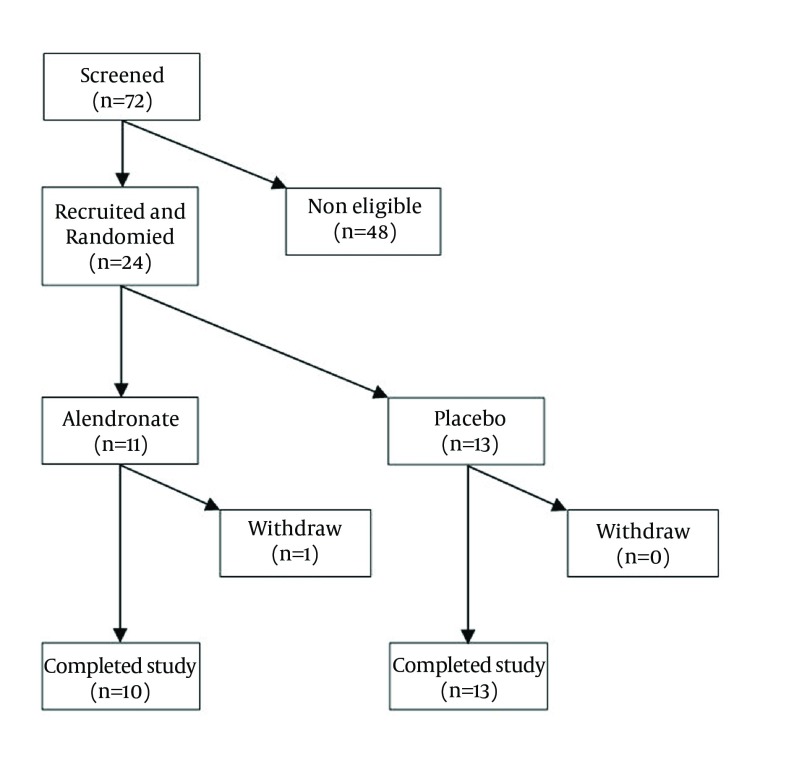
Recruitment and Enrollment of Study Participants and Outcomes

In this study, 72 patients with AS were screened for eligibility. Finally, 24 patients with early AS enrolled and were randomly allocated to the treatment and control groups. Table 1 shows the demographic and clinical characteristics of the patients and indicates no significant differences between the treatment and placebo groups at the beginning of the study. Only one patient did not complete the study (Figure 1).

**Table 1.** Demographic and Clinical Characteristics of the Studied Groups^a^

Variable	Alendronate (n = 11)	Placebo (n = 13)	P Value
**Age, y**	33.36 ± 7.8	32.85 ± 7.6	0.081
**Gender, %**			0.112
Male	7	7	
Female	4	6	
**Duration of Disease, y**	5.18 ± 4.5	5.54 ± 4.8	0.085
**ESR, mm/h**	37.8 ± 27.5	37.4 ± 25.2	0.211
**Peripheral Arthritis, No. (%)**	1 (9.1)	0	0.091
**HLA-B27, No. (%)**	9 (81.8)	12 (92.3)	0.118
**Sacroiliitis Grade**			0.092
0	0	1	
1	2	1	
2	7	2	
3	2	4	
4	0	0	
**BASDAI**	7.34 ± 2.6	7.18 ± 2.4	0.071
**BASMI**	3.20 ± 1.30	3.10 ± 1.10	0.062
**Primary Lumbar BMD, mg/cm** ^**2**^	0.99 ± 0.09	1.00 ± 0.10	0.112
**Primary Hip BMD, mg/cm** ^**2**^	0.79 ± 0.16	0.85 ± 0.15	0.061

^a^ Abbreviations: ESR, erythrocyte sedimentation rate; HLA-B27, human leukocyte antigen B27; BASDAI, Bath ankylosing spondylitis disease activity index; BASMI, Bath ankylosing spondylitis metrology index; BMD, bone mineral density.

**Figure 2.**
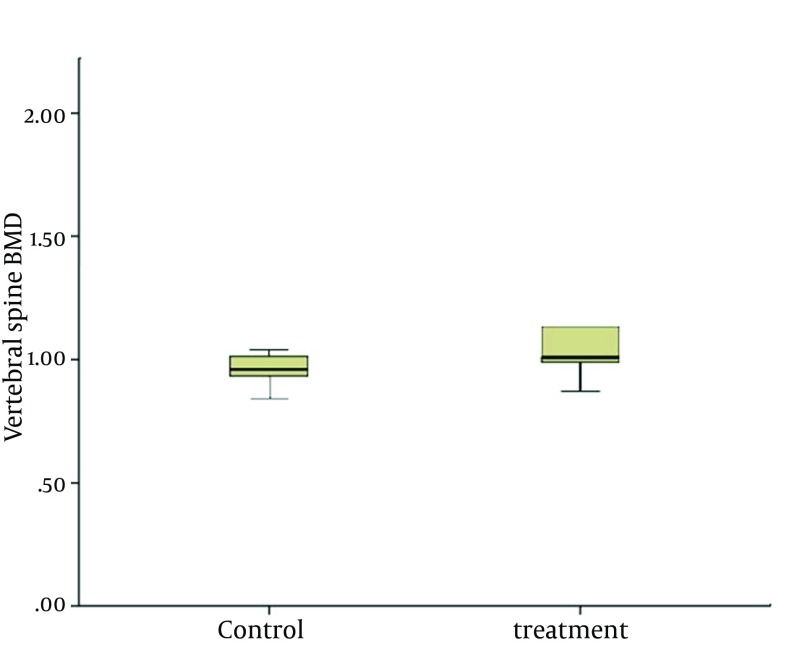
Vertebral Spines Bone Mineral Density After Intervention

**Figure 3.**
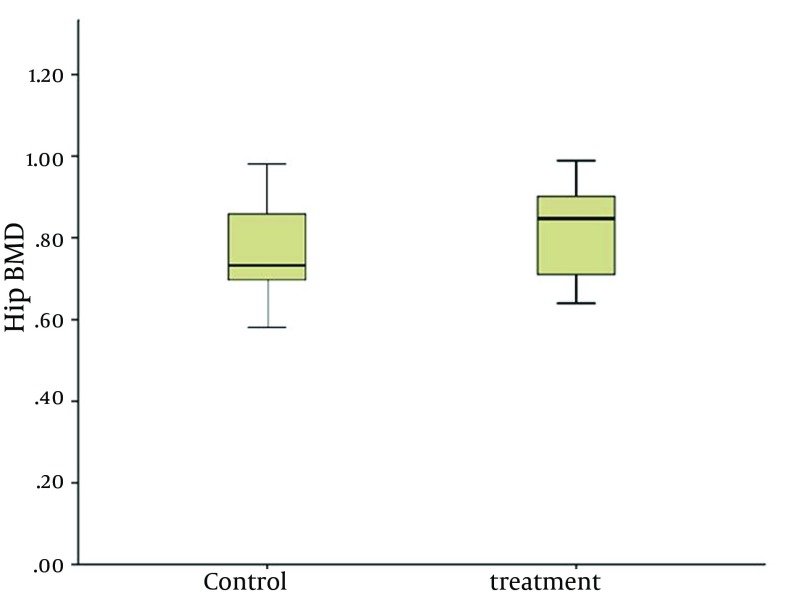
Hip Bone Mineral Density After Intervention

The difference between the lumbar and hip BMD in the treatment and control groups was insignificant after 12 months (Table 2). The effects of alendronate on vertebral and femoral BMD are illustrated in Figure 2 and 3.

**Table 2.** Bone Mineral Density in the Study Groups Before and After the Intervention^a^

Intervention	Primary Lumbar BMD, g/cm^2^	Lumbar BMD after 12 Months, g/cm^2^	Primary Hip BMD, g/cm^2^	Hip BMD After 12 Months, g/cm^2^
**Alendronate (n = 11)**	0.99 ± 0.09	1.00 ± 0.09	0.85 ± 0.15	0.84 ± 0.12
**Placebo (n = 13)**	1.00 ± 0.10	1.09 ± 0.31	0.79 ± 0.16	0.79 ± 0.15
**P Value**	0.112	0.114	0.061	0.098

^a^ Abbreviation: BMD, bone mineral density.

There was no clinically apparent vertebral and nonvertebral fracture in the treatment and control groups. No adverse events were observed.

## 5. Discussion

Bone loss is an important complication of AS. The etiology of osteoporosis in AS is multifactorial and may involves different mechanisms at different stages of the disease. The most important mechanism is inflammation (12, 23, 24). Other factors like immobilization due to pain, medications, and changes in vitamin D metabolism have also been mentioned (25). The role of reduced physical activity in patients with AS with syndesmophytes was discussed in the study by Karberg et al. (26). Genetic factors have been suspected and there is evidence that vitamin D receptor gene may contribute to BMD differences, bone metabolism, and inflammation processes in AS (27). Hormonal changes may also play a role. Franck et al. demonstrated a positive correlation between BMD at the femoral neck and serum free testosterone and free estradiol levels in men and women with AS, respectively (23). Inflammation plays an important role in the pathogenesis of bone loss in early stages of AS. In later stages of AS, mechanical factors such as decreased mobility play a more important role. Inflammatory cytokines like interleukin 1 (IL-1), interleukin 6 (IL-6) and tumor necrosis factor α (TNF-α) with the induction of RANK-RANKL system activate osteoclasts. On the other hand, osteoprotegerin (OPG), which is a potent inhibitor of RANK-RANKL system, is reduced in patients with AS (17). Maillefert et al. suggested that persistent inflammation might be an etiologic factor of bone loss in AS, and that controlling inflammatory response with anti-inflammatory drugs might reduce the bone loss in patients with AS (28). In their study, Cairns et al. showed that treatment with pamidronate pulse reduced biochemical bone turnover markers and had small beneficial effect on disease activity measured by the BASDAI (29). Maksymowych showed that intravenous pamidronate had a dose dependent anti-inflammatory effect in AS (14). However, these studies did not consider the effect of pamidronate on BMD. Up to now, to the best of our knowledge, there is no published data concerning the effects of bisphosphonates on BMD in patients with AS.

The results of our study suggest that alendronate is ineffective in preventing bone loss in early stages of AS; however, further studies with larger sample size are needed in order to generalize the results. Combination therapies might be more useful in this regard and should be examined in clinical trials.
